# Potential Role of circPVT1 as a proliferative factor and treatment target in esophageal carcinoma

**DOI:** 10.1186/s12935-019-0985-9

**Published:** 2019-10-15

**Authors:** Rongrong Zhong, Zhuozhi Chen, Ting Mo, Zimo Li, Peng Zhang

**Affiliations:** 1 Department of Geriatrics, Tianjin Medical University General Hospital, Tianjin, 300052 People’s Republic of China; 2 Tianjin Geriatrics Institute, Tianjin, 300052 People’s Republic of China; 3 The School of Life Science, Tianjin University, Tianjin, 300072 People’s Republic of China; 4 The School of Graduate, Tianjin Medical University, Tianjin, 300072 People’s Republic of China; 5 Department of Cardiothoracic Surgery, Tianjin Medical University General Hospital, Anshan Road 154, Heping District, Tianjin, 300052 People’s Republic of China

**Keywords:** Esophageal carcinoma, Cancer, circRNAs, circPVT1, Apoptosis, TE-10, miR-4663, si-RNA

## Abstract

**Background:**

Many circRNAs have been reported to play important roles in cancer development and have the potential to serve as a novel class of biomarkers for clinical diagnosis. However, the role of circRNAs in esophageal carcinoma (EC) remains unclear. In the current study, we investigated the potential role of circPVT1 in esophageal carcinoma.

**Methods:**

Quantitative real-time PCR was performed to detect circPVT1 levels. CircPVT1-specific siRNA or plasmids were used to knock down or overexpression the target RNA. Hoechst Staining was implemented to evaluate the ratio of cell apoptosis. Transwell migration assays were carried out to study the effects of circPVT1 on esophageal squamous cell carcinoma cell invasion. RegRNA 2.0 was used for bioinformatics analysis. The expression levels of Pax-4, Pax-6, PPARα and PPAR-γ were assessed using Western blot.

**Results:**

In the present study, we demonstrated a significant up-regulation of circPVT1 levels in EC tissues and cancer cell lines. The levels of circPVT1 decreased significantly when the cells were maintained to over-confluence. These results suggested a potential role for circPVT1 in cell proliferation. In addition, overexpressing circPVT1 in TE-10 cell promoted invasive ability of cancer cell. In contrast, siRNA knockdown of circPVT1 inhibited this phenomenon, leading to increased apoptosis levels of TE-10 cell. What’s more, miR-4663 had the effect of inhibiting tumor growth by downregulated Paxs and upregulated PPARs. Whereas, after the addition of circPVT1, this effect no longer worked, suggesting that circPVT1 may affect the malignancy of the tumor by affecting miRNA and regulating the levels of Paxs and PPARs.

**Conclusions:**

Collectively, our study reveals a critical role for circPVT1 in esophageal carcinoma, which may provide new insights of this circRNA as a biomarker for the diagnosis and treatment target of EC.

## Background

Esophageal cancer is one of the most aggressive squamous cell carcinomas, with more than 480,000 new cases diagnosed each year, making it the eighth most common cause of cancer death worldwide [1, 2]. It usually manifests as a high rate of lymph node metastasis, tumor invasion of adjacent tissues and organs, resulting in a large proportion of patients have metastasized before diagnosis [3, 4]. More than 80% of esophageal cancers are esophageal squamous cell carcinomas (ESCCs) [5]. Moreover, the 5-year survival rate of ESCC patients is only between 15 and 25%. Despite considerable advances in its clinical treatment, the prognosis for ESCC patients remains severe [6, 7]. The underlying mechanism of ESCC progression behind these clinical problems was still not completely clear [8] and needed to be further clarified to develop more effective therapeutic strategies [9].

CircPVT1, also known as circ6, is generated from exon 2 of the PVT1 gene and is located on chromosome 8q24, a cancer-susceptible locus. As a homologous gene of the long non-coding RNA PVT1 (human genome GRch38/hg38), this circRNA plays a critical role in regulating human physiological and pathological functions. CircPVT1 is a senescence-associated circRNA exhibiting elevated levels of expression in dividing cells to promote cell proliferation [10] and reduced levels in senescent fibroblasts to inhibit cell senescence. The Physiological functions of circPVT1 include cell proliferation, cell apoptosis and stem cell self-renewal. Whereas, the disordered expression of circRNAs leads to a variety of diseases including tumors. In recent years, circPVT1 has been extensively studied. Many studies have found that the circPVT1 is up-regulated in certain types of cancer [11], including osteosarcoma (OS) [12], breast cancer [13, 14], acute lymphoblastic leukemia (ALL), and gastric cancer (GC) [15], revealed that circPVT1 is involved in cancer cell proliferation, invasion, and metastasis. However, the role and function of circPVT1 in EC remains unclear [16].

In the current study, we aimed to determine the relationship between circPVT1 and EC. We found that circPVT1 was significantly up-regulated in the EC tissues and cell lines. In addition, we also demonstrated that overexpression of circ-PVT1 enhances the invasive ability of EC cells in vitro and downregulation of circPVT1 by siRNA could cause apoptosis of EC cells. Therefore, circPVT1 may be a potential therapeutic target of EC.

## Methods

### Human samples preparation

All patients and healthy volunteers signed an informed consent form approved by the institutional review board. We collected 20 patients with esophageal cancer and underwent complete resection at Tianjin Medical University General Hospital from 2017 to 2018. The study was approved by the Ethics Committee of Tianjin Medical University General Hospital (Ethical. NO. IRB2018-XY-034), and obtained written informed consent from all the patients. Besides, we collected blood samples from these patients prior to surgery, and other 20 age- and gender-matched healthy volunteers served as controls.

### Cell culture and treatment

The cell lines of EC109, CaES-17, TE-1, TE-10 were obtained from China Center for Type Culture Collection (CCTCC) and the Human normal esophageal epithelial cells (HEEC), HepG2, MKN45, SW60, A549 cell lines were purchased from Beijing Beina Science and technology company. The cells were cultured in DMEM (Gibco, USA) medium supplemented with 15% FBS (Gibco, USA), 100 U/mL of penicillin and 100 mg/mL of streptomycin (Hyclone, USA) at 37 °C in a humidified culture chamber (NAPCO5410, USA) supplied with 5% CO_2_ atmosphere.

### Quantitative real-time PCR (qRT-PCR) analysis

Quantitative real-time PCR (qRT-PCR) analysis was performed to detect the expression of circPVT1 [17]. Total RNA was isolated from tissues or serum using the TRIzol kit (Invitrogen, USA) and from cells using the miRNeasy Mini Kit (Qiagen, China) following to the manufacture’s guide respectively. 2 mg RNA of every sample was then incubated at 37 °C for 20 min with or without RNase R (3 U/mg, Epicentre Technologies, USA) and RNeasy MinElute Cleanup Kit (Qiagen, China) was used for RNA clean and concentration. Quantitative real-time PCR (qRT-PCR) analysis was performed to detect the expression of circPVT1 using the QuantiNova SYBR Green RT-PCR Kit (QIAGEN 208152) on the DSX System (Thermo Lab system) in accordance with the instructions. The expression of circPVT1 was normalized to GAPDH. The PCR primers were shown as follows: circPVT1 forward primers: 5′-GGTTCCACCAGCGTTATTC-3′, reverse primers: 5′-CAACTTCCTTTGGGTCTCC-3′; PVT1 forward primers: 5′-TTCAGCACTCTGGACGGACTT-3′, reverse primers: 5′-TATGGCATGGGCAGGGTAG-3′; GAPDH forward primers: 5′-AGAAGGCTGGGGCTCATTTG-3′, reverse primers: 5′-AGGGGCCATCCACAGTCTTC-3′.

### Cell transfection

Three kinds of siRNA sequences were designed for the circPVT1 and the sequence of si-circPVT1-1 was 5′-UGGGCUUGAGGCCUGAUCU-3′, si-circPVT1-2 was 5′-CUGUCAGCUGCAUGGAGCUUCGU-3′, si-circPVT1-3 was 5′-GCUUGAGGCCUGAUCUUUU-3′ and the relative si-NC sequence was 5′-AAUUCUCCGAACGUGUCACGU-3′.

The control plasmid and the circPVT1 overexpression plasmid were purchased from Biosyntech (Suzho, China). The sequences of circPVT1 and the control vector were provided in Additional file 1: Figures S1.

Cells were seeded in 12-well plates and the transfection were performed when 60–80% of cell confluency. Lipofectamine 2000 was used for the transfection according to the manufacture’s guide. All reagents were diluted and gently mixed and then incubated for 20 min at room temperature. Thereafter, the cells were added together with the mixture and incubated for another 4–6 h. TE-10 cells were collected 48 h after transfection, and the total RNA was isolated for RT-PCR to detected the expression level of CircPVT1.

### Apoptosis experiments

In the apoptosis assay, transfection was carried out to TH-10 cells with si-RNA or si-NC and the cells were cultured for another 48 h. Then Hoechst staining was performed using the Hoechst Staining Kit (Beyotime Biotechnology company, China) according to the manufacturer’s instructions. To obtain an unbiased count, cells were blindly scored without knowledge of previous treatment. Uniformly stained nuclei were scored as healthy, viable cells. Condensed or fragmented nuclei were scored as apoptotic.

### Western blotting

Western blotting was performed using standard procedures. Briefly, Cells were collected and rinsed with PBS. The total protein was extracted by addition of 2% sodium dodecyl sulfate (SDS), 125 mM Tris (pH 6.8) buffer. Lysates were sonicated and the protein was quantified by BCA Protein Assay Kit (Beyotime Biotechnology, China). Then the protein was separated by 10% sodium dodecyl sulfate polyacrylamide gel electrophoresis (SDS-PAGE) and transferred onto a polyvinylidene difluoride (PVDF) membrane (Millipore, USA). The membranes were blocked with 5% skim milk powder at room temperature for 1 h and probed with the adequate primary antibodies: anti- paired box protein 4 rabbit polyclonal antibody (Pax-4; 1:1000, ab42450, Abcam), anti- paired box protein 6 rabbit monoclonal antibody (Pax-5; 1:1000, ab109443, Abcam), anti- peroxisome proliferators-activated receptors rabbit polyclonal antibody α(PPARα; 1:1000, ab23673, Abcam), anti- peroxisome proliferators-activated receptors rabbit polyclonal antibody o (PPAR-ab 1:1000, ab45036, Abcam), anti- Glyceraldehyde-3-phosphate dehydrogenase rabbit monoclonal antibody (GAPDH; 1:1000, ab181602, Abcam) overnight at 4 °C. Then they were incubated with the adequate peroxidase-conjugated secondary antibodies (1:5000, ab6721, Abcam) at the dilutions recommended for 1 h. The analysis was performed using the Super-Signal chemiluminescence Western blotting detection system (Pierce, USA).

### Transwell invasion assay

For transwell invasion experiments, cells were treated as before and then transfected with control plasmid or the circPVT1 overexpression plasmid. TE-10 cells were collected, counted, and plated (1.5 × 105) into the 24-well Boyden chamber with a non-coated 8-mm pore size filter in the insert chamber (BD Falcon, Corning-Costar, USA). Cells were seeded into the insert chamber containing 0.5 ml Dulbecco’s modified Eagle’s medium/F12 media without containing FBS and allowed to migrate into the bottom chamber containing 0.5 ml of Dulbecco’s modified Eagle’s medium/F12 media containing 10% FBS. Transwell invasion assay lasted 24 h in a humidified incubator at 37 °C in 5% CO_2_. The 4,6-diamidino-2-phenylindole staining was used to counter the number of TE-10 and the average percentage of migrated cells was calculated.

### Bioinformatics analysis

RegRNA 2.0 (http://regrna2.mbc.nctu.edu.tw) were used as a widely used regulatory RNA motifs identification tool for bioinformatics analysis. FASTA format sequence were submitted and the predictive results were presented via a graphical interface. The sequence of first 3000 dp of PVT1 exon and cir-PVT1 were submitted to predict the potential targets miRNA and protein.

### Statistical analysis

All experiments were performed at minimal in triplicate. Results were presented as mean ± SEM. The statistical analyses were performed using Graph pad Prism 5.0. The numerical data were subjected to independent sample t-test and the levels of significance was set as **P < 0.01.

## Results

### 1. CircPVT1 was up-regulated in EC

**Fig. 1:**
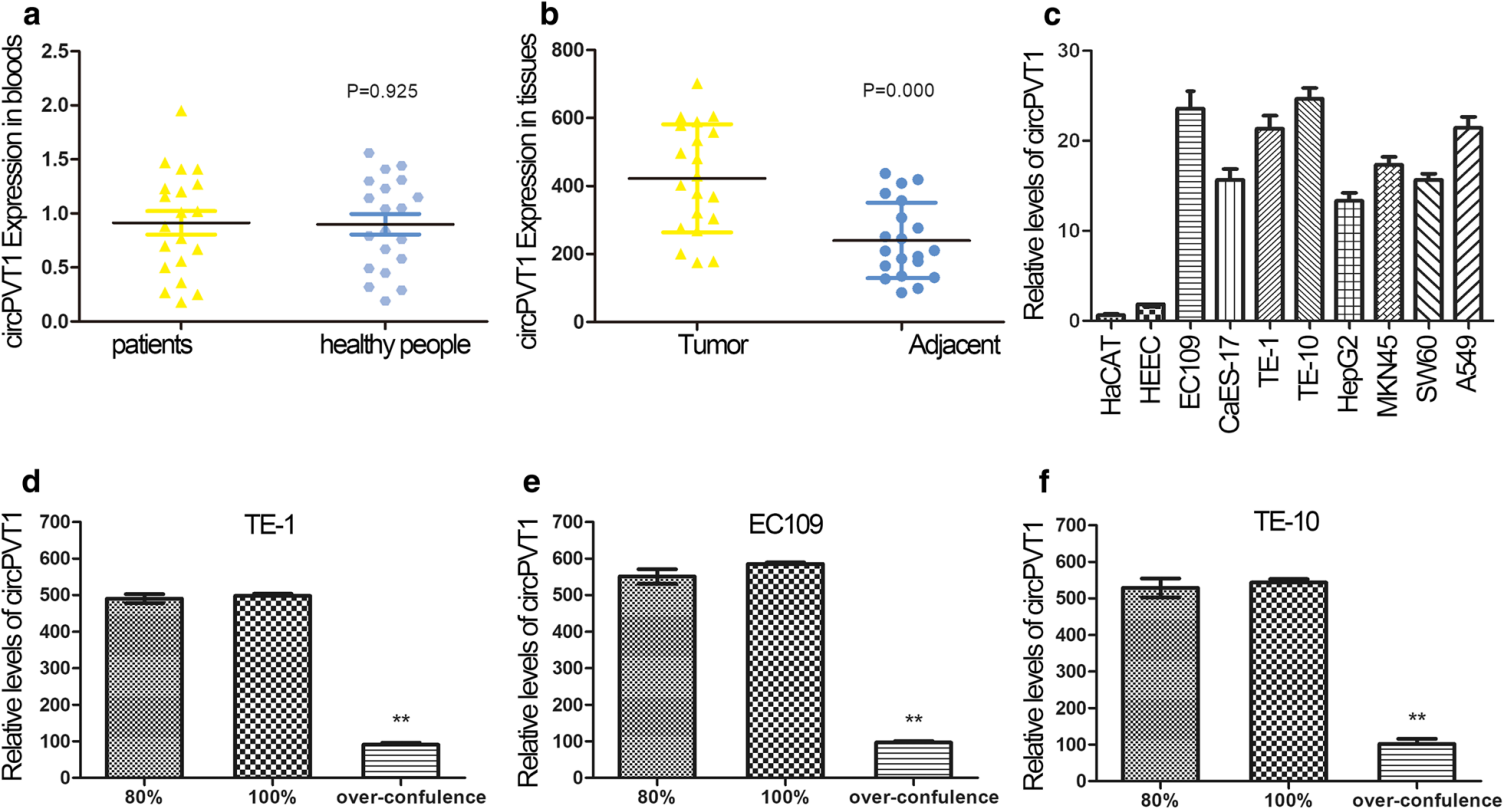
CircPVT1 was up-regulated in EC. **a** The circPVT1 expression level in blood samples was detected and there is no significant difference between EC patients and healthy people. *P *> 0.05; n = 20. **b** The circPVT1 was markedly up-regulated in EC tissues compared with the adjacent tissues. **P < 0.01; n = 20. **c** The level of circPVT1 was significantly increased in cancer cell lines, especially in EC109, TE-1 and TE-10. **P < 0.01; n = 3. **d** The circPVT1 levels decreased significantly when TE-1 cells were maintained to over-confluence. *P* < 0.01; n = 3. **e** The circPVT1 levels decreased significantly when EC109 cells were maintained to over-confluence. *P* < 0.01; n = 3. **f** The circPVT1 levels decreased significantly when TE-10 cells were maintained to over-confluence. *P* < 0.01; n = 3

We collected blood samples from 20 patients undergoing esophageal cancer surgery and 20 healthy volunteers. The expression level of circPVT1 was detected and there was no significant difference between EC patients and healthy people (Fig. 1a; P > 0.05). Then the esophagus of surgical resection was separated into tumor and para-carcinoma tissue. As shown in Fig. 1b, circPVT1 was markedly up-regulated in EC tissues compared with the adjacent tissues (Fig. 1b; P < 0.01).

In order to verify whether the highly expressed circPVT1 was produced by tumor cells, several cancerous and non-cancerous cell lines were introduced in this experiment. We observed a significant increase in CIRPVT1 levels in cancer cell lines compared with non-cancer cell lines, especially in EC109, TE-1 and TE-10 (Fig. 1c). This phenomenon indicated that the potential role of circPVT1 in cancers progression, especially in esophageal cancer.

Then EC109, TE-1 and TE-10 cell lines were cultured until over-confluence. Different stages of these three cell lines were collected and the total RNA was isolated for detection of circPVT1 levels. All three kinds of cells had high expression of this circRNA, and we did not find any significant difference between the stages before 100% confluence. However, when the cells maintained over-confluence, circPVT1 levels significantly decreased (Fig. 1d–f; P < 0.01). These results suggested that it had potential relationship between the circPVT1 expression and cell proliferation.

### 2. Silencing circ-PVT1 subsequently decreased cell survival

**Fig. 2:**
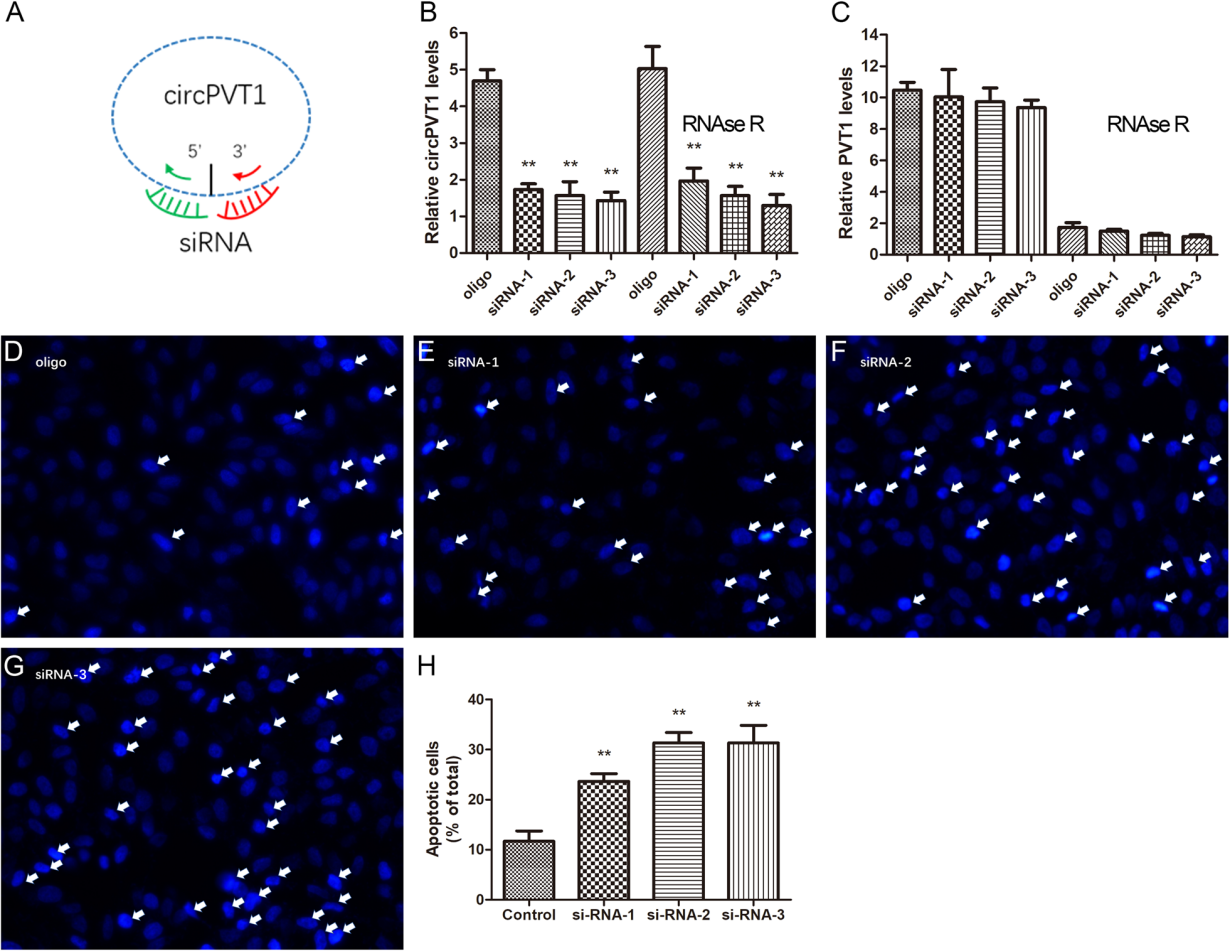
Silencing circ-PVT1 subsequently decreased cell survival. **A** The target of siRNAs were circPVT1 specifically. **B** The expression of circPVT1 was substantially decreased 48 h after treated with siRNA. **P < 0.01; n = 3. **C** The expression of linear PVT1 was not affect with siRNA. *P *> 0.05;n = 3. **D**–**G** The Hoechst staining of TE-10 cells. **H** The apoptotic cells were increased significantly after siRNA transfection to the TE-10 cells. *P* < 0.01; n = 3

To test the possibility that circPVT1 plays an important role in cell proliferation, we designed three kinds of siRNAs. The target of these siRNAs was circPVT1 specifically, which had no effect on linear PVT1 mRNA (Fig. 2A).

The TE-10 cells were transfected with these siRNAs. Forty eight hours later, TE-10 cells were collected and total RNA was isolated for RT-PCR to detect the expression levels of circPVT1. We digested linear PVT1 mRNA with RNAse-R instead of circ-PVT1. Following this, the levels of circPVT1 and linear PVT1 were detected. We found that the expression of circPVT1 was substantially decreased 48 h after treated with siRNA especially siRNA-3 (more than 69 percentage, Fig. 2B, P < 0.01) without affecting the expression of linear PVT1 (Fig. 2B, C).

To examine the phenotypic effects of these siRNAs, apoptosis of TE-10 cells was analyzed by Hoechst staining. The number of cells with apoptotic morphology appearing condensed or fragmented nuclei was counted. As shown in Fig. 2E–G, the positive cells significantly increased after siRNA was transfection to the TE-10 cells (23.67% ± 1.53% for si-RNA-1, 31.33% ± 2.08% for si-RNA-2, 31.66% ± 3.51% for si-RNA-3 respectively) compared to the control group (11.67% ± 2.08%, Fig. 2D). The number of positive cells was up-regulated by siRNAs, indicating that silencing circ-PVT1 subsequently reduced the survival ability of cancer cells (Fig. 2H).

Taken together, these data suggested that circPVT1 knockdown by siRNA might partially impeded the proliferation of EC cells and leaded to apoptosis in vitro.

### 3. Overexpression of circ-PVT1 enhanced the invasive ability of EC cells in vitro

**Fig. 3:**
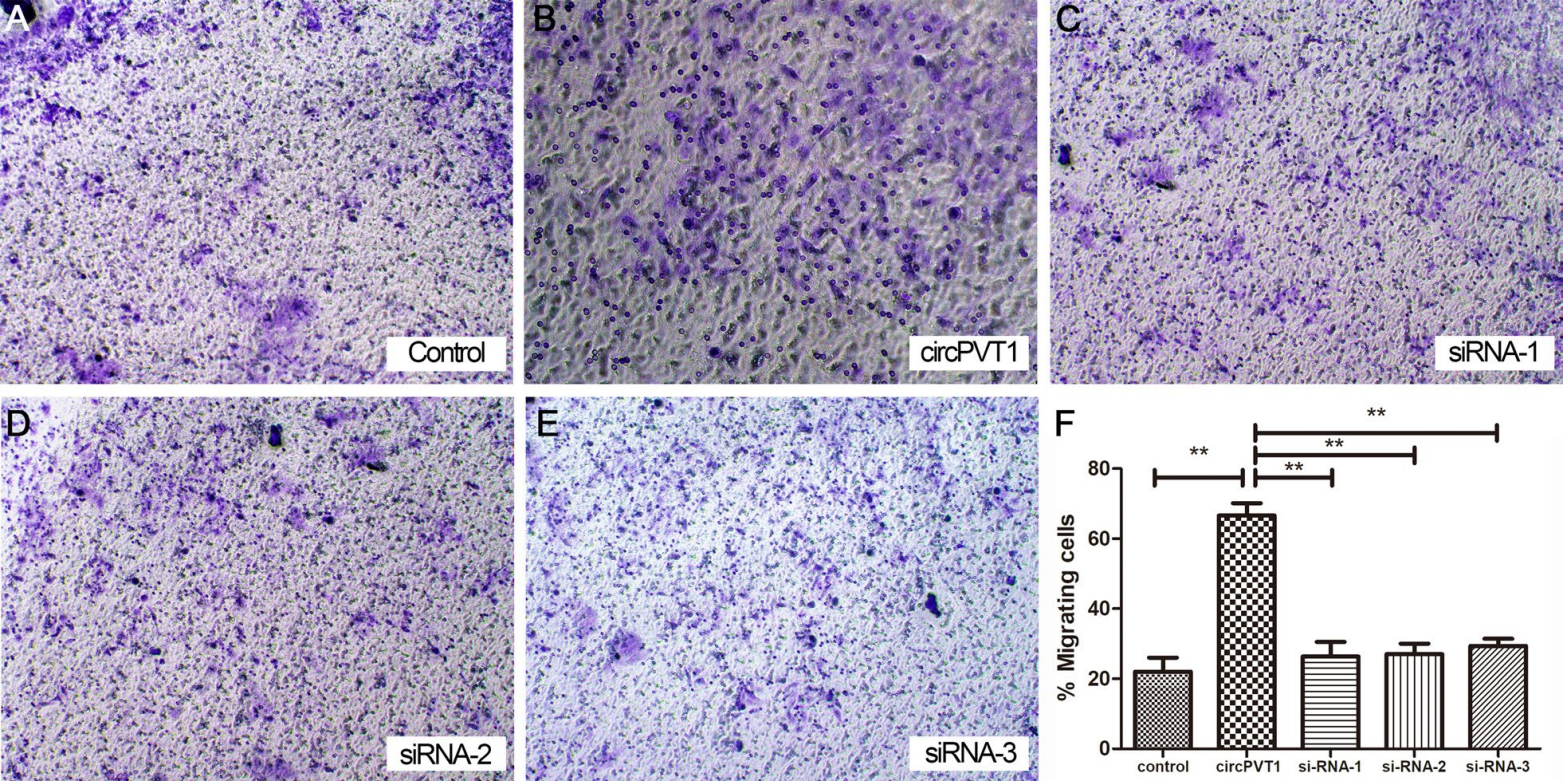
Overexpression of circ-PVT1 enhanced the invasive ability of EC cells in vitro. The transwell invasion assay was used to judge the invasiveness of TE-10 cell. **A** Control; **B** CircPVT1 over-expression; **C**, **D** treated with siRNAs. **D** Overexpression of circ-PVT1 can increase the invasive ability of TE-10 cells, and this phenomenon disappeared after knockdown of circ-PVT by siRNA. **P < 0.01; n = 3

The invasive ability of tumors is closely related to the degree of malignancy. To determine whether higher levels of circPVT1 expression would enhance tumor aggressiveness, we designed overexpression plasmids and transfected them into TE-10 cells. Then the transwell invasion assay was used to determine the invasiveness of such cancer cells. The results showed that compared with control group, the number of migrating cells was significantly increased (over 3.03-fold) after overexpressing of circPVT1 (Fig. 3A; P < 0.01).

To confirm whether this enhanced invasive ability was caused by over-expressed circPVT1, the overexpression plasmid was transfected wi siRNA and it was found that the invasive ability of tumor cells was inhibited and returned to the level of the control group (Fig. 3B; P < 0.01). In summary, overexpression of circPVT1 can increase the invasiveness of TE-10 cells, and this phenomenon disappeared after knockdown of circ-PVT by siRNA.

### 4. Prediction of the circRNA/miRNA interaction and the Potential mechanism

CircPVT1 plays an important role in the regulation of cellular proliferation and invasion during tumor progression, but how its regulatory mechanism works is still unclear in esophageal squamous cell carcinoma.

To further explore the regulatory mechanism of circPVT1, bioinformatics analysis was used in this study. The investigation of the potential miRNAs (Additional file 2: Table S1, http://regrna2.mbc.nctu.edu.tw/Results/1549033793.all.result) and binding sites (Additional file 2: Table S2, http://regrna2.mbc.nctu.edu.tw/Results/1549034097.all.result) were performed, which were based on the sequence of the first exon of the PVT1 gene. In addition, potential miRNAs that bind to circPVT1 were also investigated (Additional file 2: Table S3, http://regrna2.mbc.nctu.edu.tw/detection_output.ph) and hsa-miR-4633 was selected to construct the over expressional plasmid.

**Fig. 4:**
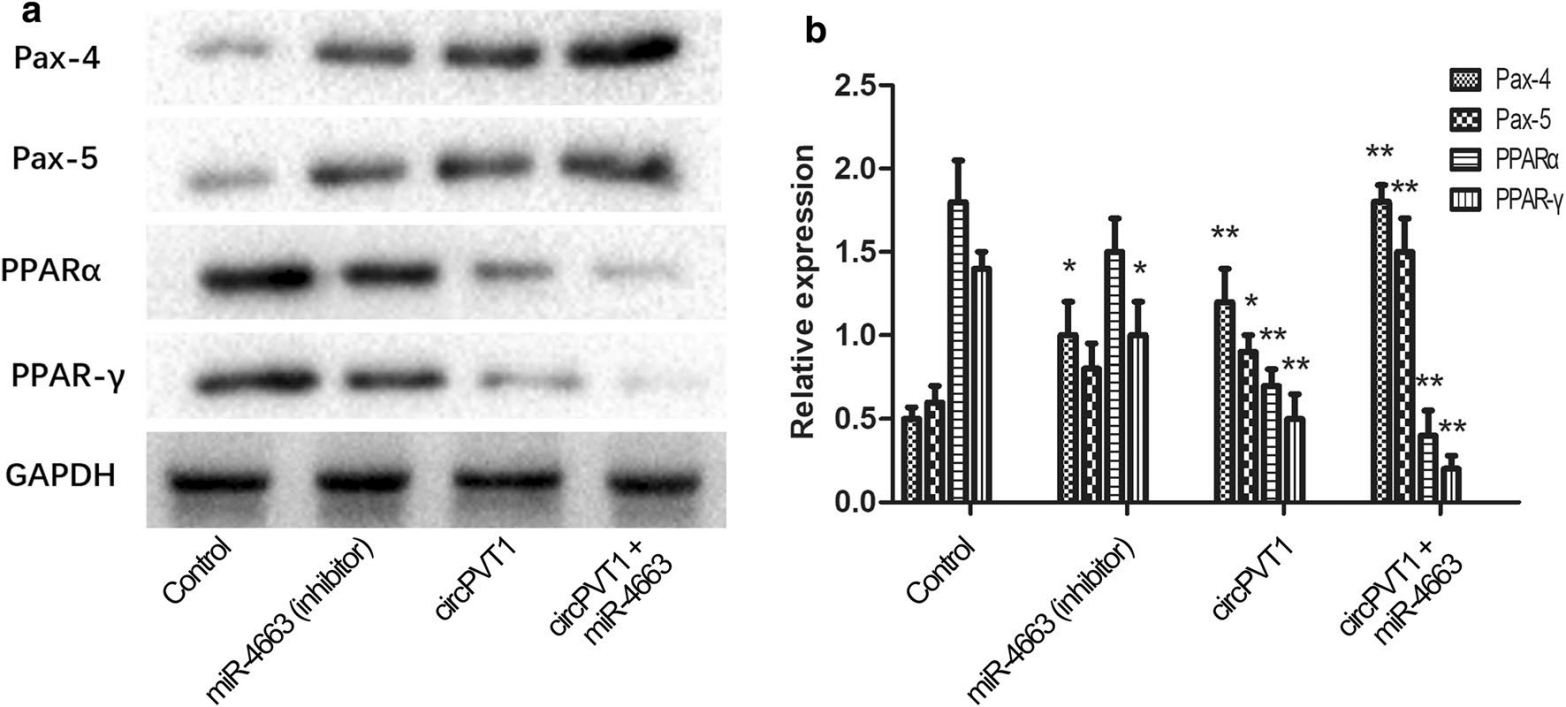
The circRNA/miRNA interaction and the Potential mechanism. The transwell invasion assay was used to judge the invasiveness of TE-10 cell. **a** Then protein levels of paired box genes (Pax-4 and Pax-6) were examined by western blotting; **b** The statistics of the western blotting. **P < 0.01; n = 3

Then protein levels of paired box genes (Pax-4 and Pax-6) were examined by western blotting, which were considered to promote tumor growth. Besides,peroxisome proliferators-activated receptors (PPARs, PPARα and PPAR-γ), which inhibit the growth of tumor were also detected. The results showed that both Pax-4 and Pax-6 were downregulated when miR-4663 was overexpressed in TE-10 cell (Fig. 4a). On the other hand, PPAR-α and PPAR-γ were up-regulated simultaneously (Fig. 4a). These experiments showed that miR-4663 had the inhibitory effect on tumor growth. Furthermore, when circPVT1 was also translated into the miR-4663 overexpression cell line, Paxs increased and PPARs decreased significantly compared to the control or circPVT1 only group, which indicated that the inhibition of tumor by miR-4663 no longer functions after the addition of circPVT1 (Fig. 4a).

Taken together, circPVT1 may affect the malignant degree of tumor by affecting miRNA and regulating the levels of Paxs and PPARs.

## Discussion

Esophageal cancer is one of the most aggressive squamous cell carcinomas, metastasis and recurrence had been the major obstacles to the clinical treatment of EC [17–19]. The prognosis of patients with ESCC is very poor, although the treatment has made great progress. Therefore, the mechanism of ESCC progression and more effective therapeutic strategies needed to be further clarified. Some studies have shown that circRNAs play an important role in regulating physiological or pathological functions [20, 21]. In recent years, there is increasing evidence that the expression of circRNAs may be associated with tumor genesis or tumor type-specific identification. They may play important role in regulation tumor cells proliferation [22–25]. However, the research on circRNAs is still in its infancy and the practical application of circRNAs as a biomarker for diagnosis or target for therapy in clinics continue to exist a long road.

In fact, many circRNAs have been reported as biomarkers for tumor diagnosis or therapy [26, 27]. For example, by studying more than 100 gastric cancer tissues with paired adjacent no tumorous tissues and plasma, hsa-circ-002059 was found to be down-regulated in gastric cancer tissues and plasma [28]. In another study, the circRNA hsa_circ_0004018 was down-regulated in hepatocellular carcinoma compared to para-tumorous tissue and correlated with decreased serum alpha-fetoprotein level, as well as tumor diameter, differentiation and stage [29]. Tumor-specific circRNA candidates were screened in lung adenocarcinoma tissue by microarrays and 59 circRNAs were found to be regulated (39 up-regulated and 20 down-regulated) [30]. Among them, hsa_circ_0013958 was further confirmed to be closely related to TNM stage and lymph node metastasis positively. These data suggested that circRNAs have the developed potential to be biomarkers or/and therapeutic targets for the diagnosis and progression of cancers. However, there are few studies on circRNA and EC. Reliable circRNA biomarker for EC diagnosis and treatment were still lack.

Several published evidence implicated that circPVT1 (hsa_circ_0001821) was a senescence suppressor and proliferative factor in aspects of cancer pathophysiology [31]. This circRNA, derived from exon 3 of the PVT1, is located on chromosome 8q24 (chr8:128902834-128903244) [10]. CircPVT1 has flanks two long introns (35269 bp and 41466 bp), which contains many Alu repeats. In humans, as a long intergenic noncoding RNA (lincRNA), Pvt1 oncogene is homologous to the mouse plasmacytoma variant translocation gene (Pvt1). In recent reports, PVT1 RNA played an important role in human cancer by regulating the protein stability of important oncogenes, including the c-Myc oncogene [32–34].

In the present study, we first examined the expression of circPVT1 in EC tissues using qRT-PCR and found that circPVT1 was distinctly up-regulated in the EC tissues compared with the para-carcinoma tissue. We found that circPVT1 expression in cancer cell lines were higher than that of HaCAT or HEEC cells, especially EC cells. Then, we further detected the relationship between circPVT1 expression level with the survival ability and malignancy degree of tumors. The result showed that circPVT1 knockdown by siRNA might partly impeded the proliferation of EC cells and leaded to apoptosis in vitro, overexpression of circ-PVT1 can increase the invasive ability of TE-10 cells, and this phenomenon disappeared after knockdown of circ-PVT. Bioinformatics analysis was used to investigation of the potential miRNAs associated with circPVT1. Western blot confirmed the role of miR-4663 in EC cells, and non-coding RNA circPVT1 can regulate the degree of malignancy of EC cells by affecting the expression of Paxs [35–37] and PPARs [38, 39].

## Conclusions

In summary, the current study demonstrated that circPVT1 is up-regulated in tissues and EC cell lines, which is associated with poor prognosis and may be a potential diagnostic biomarker for EC. In addition, it was possible that circPVT1 play a biological role by regulating the expression of Paxs and PPARs. Our findings provide new insights into the role of circPVT1 as a biomarker for the diagnosis and treatment target for EC.

## Supplementary information

**Additional file 1: Figures S1.** The sequences of circPVT1 and the control vector.

**Additional file 2: Table S1.** The potential miRNAs. **Table S2.** Binding sites of the potential miRNAs. **Table S3.** Investigation of the potential miRNAs binding with circPVT1.

## Data Availability

All data generated or analyzed during this study are included in this published article [and its additional information files].

## Abbreviations

EC: esophageal carcinoma
ESCCs: esophageal squamous cell carcinomas
OS: osteosarcoma
ALL: acute lymphoblastic leukemia
GC: gastric cancer
CCTCC: China Center for Type Culture Collection
HEEC: human normal esophageal epithelial cells
qRT-PCR: quantitative real-time PCR
Pax: paired box protein
PPAR: peroxisome proliferators-activated receptors
