# Supplementary material for: Potential Role of circPVT1 as a proliferative factor and treatment target in esophageal carcinoma
Source: Cancer Cell Int. 2019 Oct 15;19:267. doi: 10.1186/s12935-019-0985-9 (PMC6794789; doi:10.1186/s12935-019-0985-9)
Supplement: Supplementary file 1 — Additional file 1: Figures S1. The sequences of circPVT1 and the control vector. [file 12935_2019_985_MOESM1_ESM.docx]

Sequence of circPVT1

>8 dna:chromosome chromosome:GRCh37:8:128902834:128903244:1 GGCCTGATCTTTTGGCCAGAAGGAGATTAAAAAGATGCCCCTCAAGATGGCTGTGCCTGTCAGCTGCATGGAGCTTCGTTCAAGTATTTTCTGAGCCTGATGGATTTACAGTGATCTTCAGTGGTCTGGGGAATAACGCTGGTGGAACCATGCACTGGAATGACACACGCCCGGCACATTTCAGGATACTAAAAGTGGTTTTAAGGGAGGCTGTGGCTGAATGCCTCATGGATTCTTACA GCTTGGATGTCCATGGGGGACGAAGGACTGCAGCTGGCTGAGAGGGTTGAGATCTCTGTTTACTTAGATCTCTGCCAACTTCCTTTGGGTCTCCCTATGGAATGTAAGACCCCGACTCTTCCTGGTGAAGCATCTGATGCACGTTCCATCCGGCGCTCAGCTGGGCTTGAG

Sequence of mock control：

>8 dna:chromosome chromosome:GRCh37:8:128844666:128845076:1 GTATCATCAACAGGCATTTTTACTGTAGTACTTAATGTCATGCCTGGTTCAGCAAGTGGTTTTGTGACTGATTTAGCAAATAAGCATTCTCTGAAAACACAGTGAGTGAGTTCTATGTCACTAGAGGTAATCAAGAAGAGATCAAATTCATGCACATGGCCGGGTGTGGTGGCTCACGCCTGTAATCCCAGCACTTTGGGAGGCCAAGGCGGGCGGATCACGAGGTCAGGAGATAGAGAC CATCCTGGCTAACATGGTGAAACCCCATCTCTACTAAAAATACAAAAAATTAGCCGGGCGTGGTGGCGGGTGCCTGTAGTCCCAGCTACTTGGGAGGCTGAGGCAGGAGAATGGTGTGAACCCGGGAGGCGGAGCTTGCAGTGAGCTGAGATCGCACCACTCCACTCCAGC

**Primers used for the qRT-PCR and siRNA related sequence**

| circPVT1 | F: 5‘-GGTTCCACCAGCGTTATTC-3' |
| --- | --- |
|  | R: 5‘-CAACTTCCTTTGGGTCTCC-3' |
| PVT1 | F: 5‘-TTCAGCACTCTGGACGGACTT-3' |
|  | R: 5‘-TATGGCATGGGCAGGGTAG -3' |
| GAPDH | F: 5‘-AGAAGGCTGGGGCTCATTTG-3' |
|  | R: 5‘-AGGGGCCATCCACAGTCTTC-3' |
| U6 | F: 5‘-AGGGGCCATCCACAGTCTTC-3' |
|  | R: 5‘-AACGCTTCACGAATTTGCGT-3' |
| circ-PRKCI siRNA-1 | 5'-UGGGCUUGAGGCCUGAUCU-3' |
| circ-PRKCI siRNA-2 | 5'-CUGUCAGCUGCAUGGAGCUUCGU-3' |
| circ-PRKCI siRNA-3 | 5'-GCUUGAGGCCUGAUCUUUU-3' |
| relative si-NC | 5'-AAUUCUCCGAACGUGUCACGU-3' |
