# Supplementary material for: Potential Role of circPVT1 as a proliferative factor and treatment target in esophageal carcinoma
Source: Cancer Cell Int. 2019 Oct 15;19:267. doi: 10.1186/s12935-019-0985-9 (PMC6794789; doi:10.1186/s12935-019-0985-9)

**Table S1. The potential miRNAs.**

Motif Type Motif Name Position Length Sequence

open reading frame (ORF) ORF_0 1213 ~ 1458 246 atgcagaggtttctcatcatcttctttttatattttaaaagatggatgacaactagatgccaactgcagctcaggcacctgcccagtccctctcagagccgggcacaaaacagacacctgtctagcccctcacagagagccaggcacaaaacagacacctgtccagcccctcacagagagccaggcacaaagcggagacttgtccagctcctggcaaggagccaggcacaaaagagatgcccatga

open reading frame (ORF) ORF_1 2185 ~ 2541 357 atgcagatagaacaagataaccacatcccacatgggaggccagtgtccacttgctcctttggaggaactactgggcatttaagggactcgttgaacacactctgtaggtcaactctgcccggagtggagggagctgcgcagaccgctggccctctgcacgggtttgcaactcggcttctggaaacagcgggagacgtcgaaggaggcgagaaggtgcgcgcgcccttctcaccaggccggcggatgctgggtggtacccaaagaggcgctcagatcactggagctggagctcagtcggcggtcttctgcgggaactgcacctatcccggagcatctctggccctcctatttcactga

Transcriptional regulatory motif Sp1 2861 ~ 2870 10 cgggcgggga

Transcriptional regulatory motif Evi-1 2196 ~ 2204 9 acaagataa

Transcriptional regulatory motif ATF 1712 ~ 1725 14 ttgtgacgtccttc

Transcriptional regulatory motif ATF 2965 ~ 2978 14 ccgtgacgtcacgg

Transcriptional regulatory motif CREB 1715 ~ 1722 8 tgacgtcc

Transcriptional regulatory motif CREB 2968 ~ 2975 8 tgacgtca

Transcriptional regulatory motif ATF2:c-Jun 2968 ~ 2975 8 tgacgtca

Transcriptional regulatory motif NF-kappaB_(p50) 2662 ~ 2671 10 ggggattccc

Transcriptional regulatory motif YY1 728 ~ 744 17 agactccatttaaaaac

Transcriptional regulatory motif Evi-1 2189 ~ 2203 15 agatagaacaagata

Transcriptional regulatory motif MZF1 1645 ~ 1657 13 agaagaggggcaa

Transcriptional regulatory motif Ik-1 2562 ~ 2574 13 aagtgggaagacc

Transcriptional regulatory motif Ik-2 2562 ~ 2573 12 aagtgggaagac

Transcriptional regulatory motif FOXD3 1506 ~ 1517 12 aattgttttttc

Transcriptional regulatory motif HNF3beta 797 ~ 811 15 caaaatgtttatttt

Transcriptional regulatory motif Pax-5 497 ~ 524 28 ctttgggaagctgaagcgggtggatcac

Transcriptional regulatory motif RORalpha1 954 ~ 966 13 gcatcaaggtcaa

Transcriptional regulatory motif CREB 1713 ~ 1724 12 tgtgacgtcctt

Transcriptional regulatory motif CREB 2966 ~ 2977 12 cgtgacgtcacg

Transcriptional regulatory motif CREB 1713 ~ 1724 12 tgtgacgtcctt

Transcriptional regulatory motif CREB 2966 ~ 2977 12 cgtgacgtcacg

Transcriptional regulatory motif ATF2 1713 ~ 1724 12 tgtgacgtcctt

Transcriptional regulatory motif ATF2 2966 ~ 2977 12 cgtgacgtcacg

Transcriptional regulatory motif c-Myb 1036 ~ 1045 10 atcaactgtc

Transcriptional regulatory motif Sp1 2859 ~ 2871 13 ggcgggcggggac

Transcriptional regulatory motif AhR:Arnt 1809 ~ 1827 19 ggcgctggcgtggtggctc

Transcriptional regulatory motif PPARalpha:RXRalpha 953 ~ 972 20 agcatcaaggtcaaagttga

Transcriptional regulatory motif AML1a 77 ~ 82 6 tgtggt

Transcriptional regulatory motif HFH3_(FOXI1) 799 ~ 811 13 aaatgtttatttt

Transcriptional regulatory motif Freac-3 76 ~ 91 16 atgtggtaaataaata

Transcriptional regulatory motif Freac-7 2118 ~ 2133 16 aataaataaataaaat

Transcriptional regulatory motif HFH8_(FOXF1A) 799 ~ 811 13 aaatgtttatttt

Transcriptional regulatory motif Pax-3 2965 ~ 2985 21 ccgtgacgtcacgggcaaccc

Transcriptional regulatory motif ZF5 2395 ~ 2407 13 aaggtgcgcgcgc

Transcriptional regulatory motif ZF5 2397 ~ 2409 13 ggtgcgcgcgccc

Transcriptional regulatory motif ZF5 2687 ~ 2699 13 ctccggcgcgctt

Transcriptional regulatory motif ATF 1714 ~ 1725 12 gtgacgtccttc

Transcriptional regulatory motif ATF 2967 ~ 2978 12 gtgacgtcacgg

Transcriptional regulatory motif GABP 1554 ~ 1565 12 acgggaagagat

Transcriptional regulatory motif GATA-1 318 ~ 327 10 gctgataaaa

Transcriptional regulatory motif GATA-1 2197 ~ 2206 10 caagataacc

Transcriptional regulatory motif Pax-4 1740 ~ 1751 12 aacccccacccc

Transcriptional regulatory motif Pax-4 587 ~ 616 30 aaaaattagcagggcatggtggcgcacgcc

Transcriptional regulatory motif HNF4alpha1 959 ~ 973 15 aaggtcaaagttgag

Transcriptional regulatory motif AREB6 1365 ~ 1376 12 agacacctgtcc

Transcriptional regulatory motif FOXJ2 737 ~ 754 18 ttaaaaacaaacaaacaa

Transcriptional regulatory motif FOXJ2 741 ~ 758 18 aaacaaacaaacaaacaa

Transcriptional regulatory motif FOXJ2 745 ~ 762 18 aaacaaacaaacaaacaa

Transcriptional regulatory motif FOXJ2 749 ~ 766 18 aaacaaacaaacaaacaa

Transcriptional regulatory motif FOXJ2 753 ~ 770 18 aaacaaacaaacaaacaa

Transcriptional regulatory motif FOXJ2 757 ~ 774 18 aaacaaacaaacaaacaa

Transcriptional regulatory motif E2F-1 2757 ~ 2764 8 cttggcgg

Transcriptional regulatory motif Zic1 2432 ~ 2440 9 tgggtggta

Transcriptional regulatory motif Zic3 2432 ~ 2440 9 tgggtggta

Transcriptional regulatory motif FAC1 96 ~ 109 14 aaacacaacaattc

Transcriptional regulatory motif PPARgamma:RXRalpha 956 ~ 976 21 atcaaggtcaaagttgagtga

Transcriptional regulatory motif ATF3 1712 ~ 1725 14 ttgtgacgtccttc

Transcriptional regulatory motif ATF3 2965 ~ 2978 14 ccgtgacgtcacgg

Transcriptional regulatory motif ATF4 2966 ~ 2977 12 cgtgacgtcacg

Transcriptional regulatory motif LXRalpha:RXRalpha 956 ~ 970 15 atcaaggtcaaagtt

Transcriptional regulatory motif LXRalpha:RXRalpha 1126 ~ 1140 15 ctcaaggtcacagcc

Transcriptional regulatory motif LXRalpha:RXRalpha 2286 ~ 2300 15 ctgtaggtcaactct

Transcriptional regulatory motif ATF1 2965 ~ 2975 11 ccgtgacgtca

Transcriptional regulatory motif ETF 2857 ~ 2863 7 gcggcgg

Transcriptional regulatory motif ETF 2909 ~ 2915 7 gcggcgg

Transcriptional regulatory motif TFII-I 1587 ~ 1595 9 agagggagg

Transcriptional regulatory motif ZF5 1808 ~ 1815 8 gggcgctg

Transcriptional regulatory motif ZF5 2794 ~ 2801 8 gcgcgcgg

Transcriptional regulatory motif SREBP-1 1746 ~ 1752 7 cacccca

Transcriptional regulatory motif AML1 77 ~ 82 6 tgtggt

Transcriptional regulatory motif HNF3 739 ~ 751 13 aaaaacaaacaaa

Transcriptional regulatory motif HNF3 743 ~ 755 13 acaaacaaacaaa

Transcriptional regulatory motif HNF3 747 ~ 759 13 acaaacaaacaaa

Transcriptional regulatory motif HNF3 751 ~ 763 13 acaaacaaacaaa

Transcriptional regulatory motif HNF3 755 ~ 767 13 acaaacaaacaaa

Transcriptional regulatory motif HNF3 759 ~ 771 13 acaaacaaacaaa

Transcriptional regulatory motif HNF3 763 ~ 775 13 acaaacaaacaaa

Transcriptional regulatory motif SMAD 1324 ~ 1332 9 agacacctg

Transcriptional regulatory motif SMAD 1365 ~ 1373 9 agacacctg

Transcriptional regulatory motif AP-2 2849 ~ 2864 16 cgcaccgggcggcggg

Transcriptional regulatory motif AP-2 2856 ~ 2871 16 ggcggcgggcggggac

Transcriptional regulatory motif AP-2 2908 ~ 2923 16 cgcggcgggccgggcc

Transcriptional regulatory motif E2F 1958 ~ 1963 6 ggcgcg

Transcriptional regulatory motif E2F 2691 ~ 2696 6 ggcgcg

Transcriptional regulatory motif LEF1 963 ~ 968 6 tcaaag

Transcriptional regulatory motif FOX_factors 799 ~ 811 13 aaatgtttatttt

Transcriptional regulatory motif MYB 1038 ~ 1046 9 caactgtca

Transcriptional regulatory motif CREB 2966 ~ 2979 14 cgtgacgtcacggg

Transcriptional regulatory motif CREB 1712 ~ 1722 11 ttgtgacgtcc

Transcriptional regulatory motif CREB 2965 ~ 2975 11 ccgtgacgtca

Transcriptional regulatory motif Sp1 2861 ~ 2870 10 cgggcgggga

Transcriptional regulatory motif Sp1 2859 ~ 2871 13 ggcgggcggggac

Transcriptional regulatory motif VDR,_CAR, 2265 ~ 2285 21 aagggactcgttgaacacact

Transcriptional regulatory motif IRF 447 ~ 457 11 aaaaatgaaag

Transcriptional regulatory motif E2A 1288 ~ 1295 8 cacctgcc

Transcriptional regulatory motif E2A 1327 ~ 1334 8 cacctgtc

Transcriptional regulatory motif E2A 1368 ~ 1375 8 cacctgtc

Transcriptional regulatory motif CREB,_ATF 1714 ~ 1722 9 gtgacgtcc

Transcriptional regulatory motif CREB,_ATF 2967 ~ 2975 9 gtgacgtca

Transcriptional regulatory motif Cdx 877 ~ 894 18 aaaaaagaaattaataaa

Transcriptional regulatory motif Cdx 2094 ~ 2111 18 aataaataaataaataaa

Transcriptional regulatory motif Cdx 2098 ~ 2115 18 aataaataaataaataaa

Transcriptional regulatory motif Cdx 2102 ~ 2119 18 aataaataaataaataaa

Transcriptional regulatory motif Cdx 2106 ~ 2123 18 aataaataaataaataaa

Transcriptional regulatory motif Cdx 2110 ~ 2127 18 aataaataaataaataaa

Transcriptional regulatory motif Cdx 2114 ~ 2131 18 aataaataaataaataaa

Transcriptional regulatory motif IPF1 1064 ~ 1078 15 tctgccattagaagc

Transcriptional regulatory motif RBP-Jkappa 2561 ~ 2571 11 aaagtgggaag

Transcriptional regulatory motif WT1 2937 ~ 2945 9 ccctccccc

Transcriptional regulatory motif Kid3 1094 ~ 1098 5 ccacc

Transcriptional regulatory motif Kid3 1552 ~ 1556 5 ccacg

Transcriptional regulatory motif Kid3 1681 ~ 1685 5 ccacc

Transcriptional regulatory motif Kid3 1745 ~ 1749 5 ccacc

Transcriptional regulatory motif CKROX 2936 ~ 2944 9 cccctcccc

Transcriptional regulatory motif Nkx3-2 1780 ~ 1785 6 taagtg

Transcriptional regulatory motif CTF1 1424 ~ 1437 14 tggcaaggagccag

Transcriptional regulatory motif FOXO1 739 ~ 747 9 aaaaacaaa

Transcriptional regulatory motif GTF2IRD1-isoform2 1174 ~ 1182 9 aggattaag

Transcriptional regulatory motif ZNF333 411 ~ 415 5 ataat

Transcriptional regulatory motif BEN 2369 ~ 2376 8 cagcggga

Transcriptional regulatory motif Nanog 773 ~ 792 20 aaaacaaaacaaaagcagat

Transcriptional regulatory motif CNOT3 2920 ~ 2929 10 ggccgcgcgc

Transcriptional regulatory motif IPF1 181 ~ 186 6 cattaa

Transcriptional regulatory motif NFAT1 897 ~ 902 6 ggaaaa

Transcriptional regulatory motif NFAT1 1151 ~ 1156 6 ggaaaa

Transcriptional regulatory motif HOXA13 322 ~ 327 6 ataaaa

Transcriptional regulatory motif HOXA13 890 ~ 895 6 ataaaa

Transcriptional regulatory motif HOXA13 2127 ~ 2132 6 ataaaa

Transcriptional regulatory motif Ncx 880 ~ 896 17 aaagaaattaataaaaa

Transcriptional regulatory motif PITX2 1170 ~ 1186 17 tgaaaggattaaggtct

Transcriptional regulatory motif HOXB8 879 ~ 894 16 aaaagaaattaataaa

Transcriptional regulatory motif Pitx1 1169 ~ 1185 17 gtgaaaggattaaggtc

Transcriptional regulatory motif ERR2_(ESRRB) 1127 ~ 1138 12 tcaaggtcacag

Transcriptional regulatory motif TCF-3 842 ~ 854 13 tctttgtattttt

Transcriptional regulatory motif STAT3 2825 ~ 2840 16 gctgccgggaagcagg

Transcriptional regulatory motif DRI1 885 ~ 890 6 aattaa

Transcriptional regulatory motif p53 1037 ~ 1056 20 tcaactgtcaggacatggac

Transcriptional regulatory motif MAFA 2553 ~ 2559 7 tcagcag

Transcriptional regulatory motif OTX2 1171 ~ 1183 13 gaaaggattaagg

Transcriptional regulatory motif ING4 1094 ~ 1099 6 ccacca

Transcriptional regulatory motif AML2 2203 ~ 2210 8 aaccacat

Transcriptional regulatory motif NMYC 269 ~ 274 6 catctg

Transcriptional regulatory motif NMYC 290 ~ 295 6 catctg

Transcriptional regulatory motif NMYC 2164 ~ 2169 6 catctg

Transcriptional regulatory motif CREM 2968 ~ 2978 11 tgacgtcacgg

Transcriptional regulatory motif c-Myb 1036 ~ 1046 11 atcaactgtca

Transcriptional regulatory motif ATF-1 2968 ~ 2976 9 tgacgtcac

Transcriptional regulatory motif ATF-2 1712 ~ 1723 12 ttgtgacgtcct

Transcriptional regulatory motif ATF-2 2965 ~ 2976 12 ccgtgacgtcac

Transcriptional regulatory motif ATF-4 2969 ~ 2977 9 gacgtcacg

Transcriptional regulatory motif C/EBPalpha 418 ~ 430 13 cacttttgcaaga

Transcriptional regulatory motif NF-AT4 897 ~ 902 6 ggaaaa

Transcriptional regulatory motif NF-AT4 1151 ~ 1156 6 ggaaaa

Transcriptional regulatory motif PLAG1 2742 ~ 2757 16 cccccagtctgggccc

Transcriptional regulatory motif ERR3 958 ~ 965 8 caaggtca

Transcriptional regulatory motif ERR3 1128 ~ 1135 8 caaggtca

Transcriptional regulatory motif HTF4 1288 ~ 1294 7 cacctgc

Transcriptional regulatory motif MEF-2C 197 ~ 203 7 tattttt

Transcriptional regulatory motif MEF-2C 343 ~ 349 7 tattttt

Transcriptional regulatory motif MEF-2C 835 ~ 841 7 tattttt

Transcriptional regulatory motif MEF-2C 848 ~ 854 7 tattttt

Transcriptional regulatory motif PARP 133 ~ 138 6 tttctt

Transcriptional regulatory motif PARP 156 ~ 161 6 tttctt

Transcriptional regulatory motif PARP 334 ~ 339 6 tttctt

Transcriptional regulatory motif PARP 840 ~ 845 6 tttctt

Transcriptional regulatory motif PARP 1514 ~ 1519 6 tttctt

Transcriptional regulatory motif AML1 77 ~ 82 6 tgtggt

Transcriptional regulatory motif Cdx-1 804 ~ 809 6 tttatt

Transcriptional regulatory motif CDX-2 1239 ~ 1244 6 tttata

Transcriptional regulatory motif E2F-3 2863 ~ 2869 7 ggcgggg

Transcriptional regulatory motif ERR1 1128 ~ 1138 11 caaggtcacag

Transcriptional regulatory motif ERR3 1125 ~ 1137 13 cctcaaggtcaca

Transcriptional regulatory motif LHX3 576 ~ 581 6 attaaa

Transcriptional regulatory motif LHX3 825 ~ 830 6 attaaa

Transcriptional regulatory motif LRH-1 955 ~ 965 11 catcaaggtca

Transcriptional regulatory motif LRH-1 1125 ~ 1135 11 cctcaaggtca

Transcriptional regulatory motif pitx2 484 ~ 493 10 tgtaatccca

Transcriptional regulatory motif pitx2 1833 ~ 1842 10 tgtaatccca

Transcriptional regulatory motif SOX10 1398 ~ 1404 7 cacaaag

Transcriptional regulatory motif NFATC2 1012 ~ 1018 7 ttttcca

Transcriptional regulatory motif NR4A2 1129 ~ 1136 8 aaggtcac

Transcriptional regulatory motif CREB1 2968 ~ 2975 8 tgacgtca

Transcriptional regulatory motif SOX10 43 ~ 48 6 ctttgt

Transcriptional regulatory motif SOX10 843 ~ 848 6 ctttgt

**Table S2. Binding sites** **of the potential miRNAs.**

Motif Type Motif Name Position Length Sequence

open reading frame (ORF) ORF_0 1213 ~ 1458 246 atgcagaggtttctcatcatcttctttttatattttaaaagatggatgacaactagatgccaactgcagctcaggcacctgcccagtccctctcagagccgggcacaaaacagacacctgtctagcccctcacagagagccaggcacaaaacagacacctgtccagcccctcacagagagccaggcacaaagcggagacttgtccagctcctggcaaggagccaggcacaaaagagatgcccatga

open reading frame (ORF) ORF_1 2185 ~ 2541 357 atgcagatagaacaagataaccacatcccacatgggaggccagtgtccacttgctcctttggaggaactactgggcatttaagggactcgttgaacacactctgtaggtcaactctgcccggagtggagggagctgcgcagaccgctggccctctgcacgggtttgcaactcggcttctggaaacagcgggagacgtcgaaggaggcgagaaggtgcgcgcgcccttctcaccaggccggcggatgctgggtggtacccaaagaggcgctcagatcactggagctggagctcagtcggcggtcttctgcgggaactgcacctatcccggagcatctctggccctcctatttcactga

microRNA target sites hsa-miR-125a-5p 2597 ~ 2619 23 ggaagggtaagagggctcaggga

microRNA target sites hsa-miR-612 1276 ~ 1299 24 ctgcagctcaggcacctgcccagt

microRNA target sites hsa-miR-1271-3p 1420 ~ 1442 23 ctcctggcaaggagccaggcaca

microRNA target sites hsa-miR-2113 1343 ~ 1363 21 cacagagagccaggcacaaaa

microRNA target sites hsa-miR-2113 1384 ~ 1404 21 cacagagagccaggcacaaag

microRNA target sites hsa-miR-2113 1302 ~ 1322 21 ctctcagagccgggcacaaaa

microRNA target sites hsa-miR-2113 1423 ~ 1445 23 ctggcaaggagccaggcacaaaa

microRNA target sites hsa-miR-1304-3p 667 ~ 688 22 ggaggtggaggttgcagtgagc

microRNA target sites hsa-miR-4726-3p 2856 ~ 2877 22 ggcggcgggcggggacctgggg

microRNA target sites hsa-miR-5095 468 ~ 488 21 cacggtggctcacgcctgtaa

microRNA target sites hsa-miR-5095 1817 ~ 1837 21 cgtggtggctcacgcctgtaa

microRNA target sites hsa-miR-5095 601 ~ 621 21 catggtggcgcacgcctgtag

microRNA target sites hsa-miR-5096 546 ~ 566 21 tcctggccaacatggtgaaac

microRNA target sites hsa-miR-5096 1897 ~ 1917 21 gcctggccaacatggtgaaac

**Table S3. Investigation of the potential miRNAs binding with circPVT1**

Motif Type Motif Name Position Length Sequence

microRNA target sites hsa-miR-4663 376 ~ 402 27 gatgcacgttccatccggcgctcagct


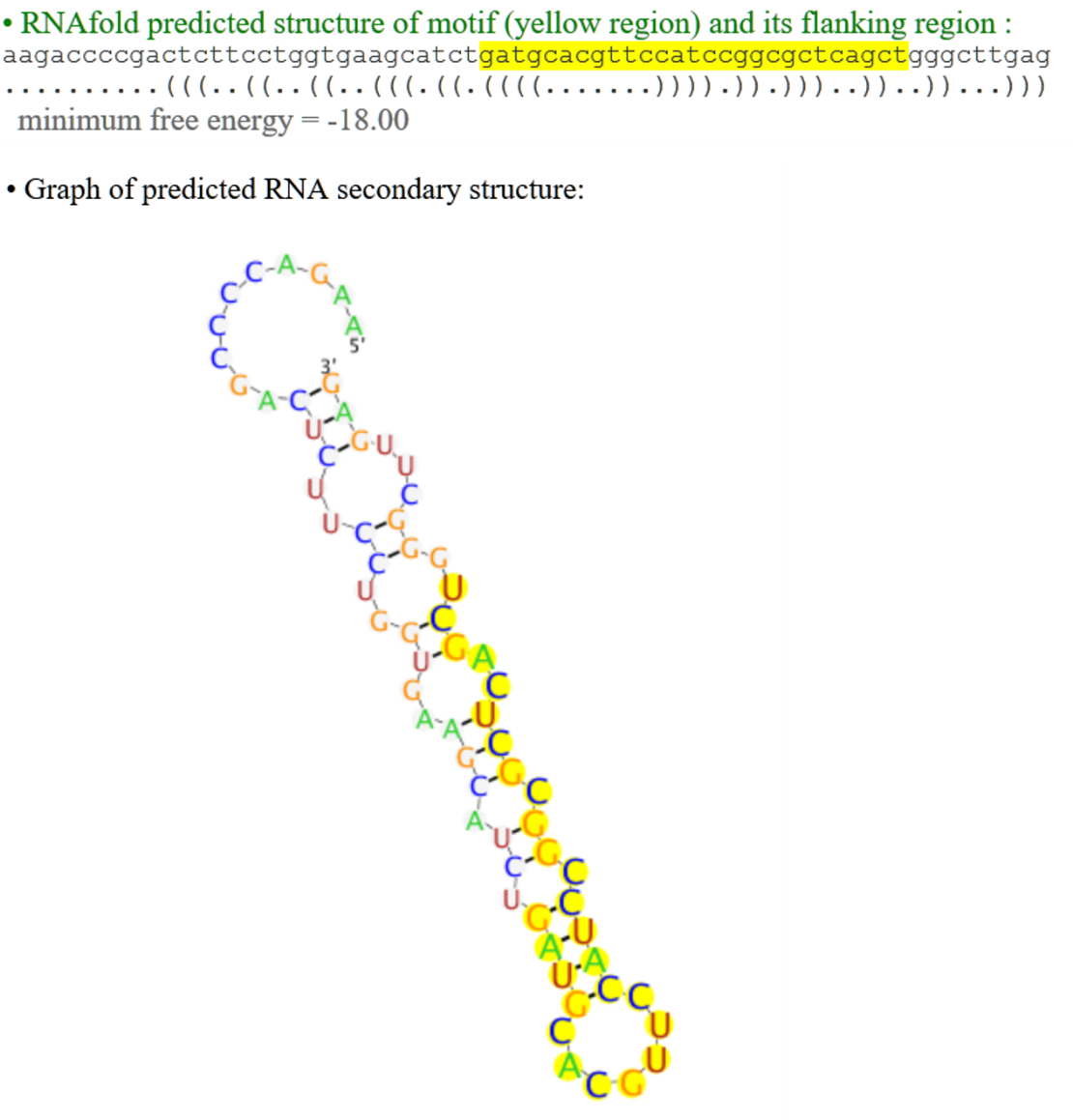

Supplement: Supplementary file 2 — Additional file 2: Table S1. The potential miRNAs. Table S2. Binding sites of the potential miRNAs. Table S3. Investigation of the potential miRNAs binding with circPVT1. [file 12935_2019_985_MOESM2_ESM.docx]
